# Barriers Toward the National Program for Prevention and Control of Diabetes in Iran: A Qualitative Exploration

**DOI:** 10.34172/ijhpm.2022.6908

**Published:** 2022-10-18

**Authors:** Sohila Sadeghi, Fatemeh Mahani, Parisa Amiri, Shahram Alamdari, Davood Khalili, Navid Saadat, Seyed Alireza Ebadi, Ali Reza Mahdavi Hazaveh, Mohammad Karim Shahrzad, Fereidoun Azizi

**Affiliations:** ^1^Research Center for Social Determinants of Health, Research Institute for Endocrine Sciences, Shahid Beheshti University of Medical Sciences, Tehran, Iran.; ^2^Department of Endocrinology & Metabolism, Internal Medicine, School of Medicine, Imam Hossein Hospital, Shahid Beheshti University of Medical Sciences, Tehran, Iran.; ^3^Obesity Research Center, Research Institute for Endocrine Sciences, Shahid Beheshti University of Medical Sciences, Tehran, Iran.; ^4^Prevention of Metabolic Disorders Research Center, Research Institute for Endocrine Sciences, Shahid Beheshti University of Medical Sciences, Tehran, Iran.; ^5^Ministry of Health and Medical Education, Center for Non-communicable Disease Control, Tehran, Iran.; ^6^Internal Medicine and Endocrinology Shohada Tajrish Medical Center, Shahid Beheshti University of Medical Sciences, Tehran, Iran.; ^7^Endocrine Research Center, Research Institute for Endocrine Sciences, Shahid Beheshti University of Medical Sciences, Tehran, Iran.

**Keywords:** Diabetes, Barriers, National Program, The NPPCD, Qualitative Study, Iran

## Abstract

**Background:** Despite the achievements of the national program for the prevention and control of diabetes (NPPCD) over the past two decades, the available evidence indicates a high prevalence of this disease in Iran. This qualitative study aims to investigate barriers to the NPPCD by pursuing the perspectives of relevant policy-makers, planners, and healthcare workers.

**Methods:** A grounded theory approach was used to analyze participants’ perceptions and experiences. Semi-structured interviews (n=23) and eight focus groups (n=109) were conducted with relevant policy-makers, planners, and healthcare workers in charge of Iran’s national diabetes management program. Of the 132 participants, ages ranged from 25 to 56 years, and 53% were female. Constant comparative analysis of the data was conducted manually, and open, axial, and selective coding was applied to the data.

**Results:** Two main themes emerged from data analysis: implementation barriers and inefficient policy-making/ planning. Insufficient financial resources, staff shortage and insufficient motivation, inadequate knowledge of some healthcare workers, and defects in the referral system were recognized as the NPPCD implementation barriers. Inappropriate program prioritizing, the lack of or poor intersectoral collaboration, and the lack of an effective evaluation system were the inefficient policy-making/planning problems.

**Conclusion:** Current results highlighted that inefficient policy-making and planning have led to several implementation problems. Moreover, the key strategies to promote this program are prioritizing the NPPCD, practical intersectoral collaboration, and utilizing a more efficient evaluation system to assess the program and staff performance.

## Background

**Key Messages**

** Implications for policy makers**

- Increasing the priority of the diabetes national program could lead to an improvement in diabetes healthcare services.
- Policy-making for a more efficient intersectoral collaboration seems essential to level up the diabetes national program.
- The national program for the prevention and control of diabetes (NPPCD) will benefit from providing new guidelines and protocols for a functional evaluation system to assess the program and staff performance.

** Implications for the public**

Current data could provide a comprehensive understanding of the national program for the prevention and control of diabetes’ (NPPCD’s) defects from the perspective of relevant policy-makers, planners, and healthcare workers in Iran. Exploring the main barriers at executive and policy-making/planning levels can improve the program and promote public health. Consequently, implementing a more effective program will assist individuals in managing diabetes and decrease its multiple complications. Additionally, our findings can be a clue for discovering deficiencies in diabetes care programs and detecting practical solutions to overcome them. Hence, countries with similar shortcomings in their national diabetes healthcare plans might benefit from the present study’s findings.

Over the past decades, the prevalence of diabetes in the world and Iran has dramatically increased.^1,2^ It has been estimated that 9.2 million of the Iranian population will have diabetes by 2030.^3^ Diabetes is a lifelong high-cost chronic disease leading to many physical, psychosocial, and economic complications, resulting in a noticeable burden for individuals, societies, and healthcare systems.^4-10^ In this regard, policy-making and planning for diabetes management have become a critical concern nationally and internationally.^6^

 The World Health Organization (WHO), In 1989, called on member states to take with implement policies and programs for diabetes management.^11^ Although most countries reported that they developed national policies, guidelines, and protocols, there was a lack of funding and implementation in some areas.^9^ As a member state of WHO, Iran was among the first countries in the Eastern Mediterranean region to operate a national diabetes management program. In 1991, a pilot project was implemented to prevent and control diabetes in three rural areas and was discontinued in 1993 due to some obstacles. The Iranian national advisory committee resumed its work in 1996, and with the support of health authorities, a national strategy was developed to manage diabetes.^12^ Subsequently, the national program for the prevention and control of diabetes (NPPCD) was designed in 1999 and initiated with a pilot study conducted in 17 medical universities. After the pilot study, the NPPCD has launched in two rural and urban phases from 2004.^13,14^ Following the WHO global package of essential non-communicable diseases,^15^ the NPPCD has continued as a subprogram within a relevant framework in Iran.

 The NPPCD has been operated at three main levels to prevent and control diabetes in Iran. The first level contains health houses that screen and evaluates high-risk individuals in rural areas by Behvarz workers (primary healthcare workers in rural areas). General practitioners and laboratory facilities are available at the second level in health centers. The third level was designed for patients needing specialized care and is located in the district hospitals. The second and third levels are responsible for detecting and controlling diabetes.^12^

 Implementing the NPPCD has led to positive results, including a gradual improvement in the quality of diabetes care and success in identifying high-risk individuals.^16^ In addition, the Behvarz workers’ healthcare services in rural areas were associated with lower fasting plasma glucose levels.^17^ However, an analysis of the NPPCD documents revealed gaps and limitations related to the healthcare and insurance system, funding shortage, and insufficient key stakeholders’ collaboration.^14^ Moreover, a qualitative study that has explored patients’ and healthcare workers’ perspectives also identified some obstacles.^18^ Nevertheless, the perceptions and experiences of policy-makers and planners in charge of the NPPCD are unknown.

 Due to the increasing prevalence of diabetes among the Iranian population and its complications, it seems barriers remain. Hence, exploring the experiences of the relevant policy-makers and planners regarding the NPPCD after 25 years of implementing the program seems critical to recognize existing problems and create a more comprehensive understanding of these limitations to program improvement. In the current study, barriers to the NPPCD were investigated from the perspective of 132 individuals selected among the highly influential policy-makers, planners, and healthcare workers in charge of the NPPCD.

## Methods

###  Participants and Data Collection 

 In order to identify the NPPCD’s barriers, the current qualitative study has investigated the perceptions and experiences of policy-makers, planners, and healthcare workers in charge of the NPPCD. The participants’ selection criteria were as follows: (1) The policy-makers and planners who have had influential roles in the health system of Iran, (2) Healthcare workers in charge of diabetes management affiliated with Shahid Beheshti University of Medical Sciences with the diversity of urban and rural areas centers in Tehran. These healthcare workers were selected for the following reasons: (1) Tehran is the most populous province in the country, with one of the highest rates of diabetes, and (2) most of the population of this province is covered by the health services of Shahid Beheshti University of Medical Sciences. Therefore, the service providers at this university had sufficient experience in managing diabetes with different social and economic groups of urban and rural populations under their auspices.

 An interview guide was developed, including a list of interview questions and a guideline for the interviewer to collect informed consent and the essential participants’ information. The mentioned guide has been provided by authors and initially tested through pre-interviews. A total of 23 in-depth interviews and eight focus groups (n = 109) were conducted between December 2019 and February 2020. Of the 132 participants, the ages ranged from 25 to 56 years, and 53% were female. Participants’ information is provided in Table.

**Table:** Study Participants

	**In-depth Interviews**	**Age (y) and Gender**	**Work Experience (y)**	**Focus Groups** **(No. of participants)**
Policy-makers/planners^a^	5	48/Female	18	6 (12-15)
		45/Female	17	
		52/Male	22	
		56/Male	23	
		49/Male	21	
Healthcare workers				2 (12-13)
Family physicians	2	38/Female	3	
		42/Male	5	
General practitioners	5	48/Female	17	
		44/Female	12	
		35/Female	6	
		45/Male	15	
		50/Male	23	
Non-communicable diseases experts	3	38/Female	10	
		43/Male	13	
		36/Male	12	
Healthcare providers	6	29/Female	5	
		33/Female	13	
		40/Female	14	
		30/Male	8	
		42/Male	16	
		45/Male	20	
Behvarz workers^b^	2	38/Female	16	
		25/Female	3	

^a^Non-communicable diseases managers, the Ministry of Health and Medical Education managers, diabetes experts, and deans of medical universities. ^b^Primary healthcare workers in rural areas.

 Purposive sampling was utilized to select participants with highly related experiences regarding the NPPCD. The policy-makers and planners included non-communicable diseases managers, the Ministry of Health and Medical Education (MoHME) managers, diabetes experts, and deans of medical universities from different provinces of Iran. The healthcare workers included non-communicable diseases experts, general practitioners, family physicians, Behvarz workers, and healthcare providers (healthcare workers in urban areas). In order to obtain the experiences of policy-makers and planners, six focus groups and five in-depth interviews were conducted. Besides, to collect information from the healthcare workers of primary and secondary care levels, 18 in-depth interviews and two focus groups were conveyed. A semi-structured guide comprising open-ended questions was asked in a private room, enabling participants to explain their perceptions and experiences sufficiently. Semi-structured interviews and focus groups were chosen as the best means to collect data. The main researcher (first author) communicated with potential participants (in their workplaces) and described the purposes as well as the process of the current research. A face-to-face interview or focus group discussion was scheduled if the participants agreed to partake in the study. Subsequently, interviews and focus groups were conducted in participants’ workplaces (offices and public healthcare centers). The participants were asked to share their perceptions and experiences regarding the barriers to the implementation of NPPCD. They were then asked about the leading causes of these obstacles.

 The main researcher conducted all interviews. Each in-depth interview lasted 25 to 45 minutes, and each focus group lasted between 1 and 3 hours. Data was collected using a voice recorder, and field notes were made during the interviews and focus groups. All the interviews were transcribed verbatim in Farsi; then, those quotations were selected by the research group, transcribed to English by the second first author, and reviewed through research team meetings to validate translation quality and conceptual equality.

###  Data Analysis 

 Data were manually analyzed and guided by a constant comparative analysis.^19^ Data were collected and analyzed simultaneously based on the grounded theory approach. Open, axial, and selective coding was applied to the current data. All transcripts were reviewed by the main researcher or at least another study team member during open coding, and data were reduced to codes. The other research team members reviewed the coded transcript; then, differences in coding were resolved during the group discussions. After comparing codes, those that were found to be conceptually similar or had a related meaning were classified into subcategories. Axial coding was done to clarify how the emergent subcategories were associated with preliminary categories. Finally, the main themes were derived from the data analysis. Analytical tools were used, including asking questions and making comparisons to find the properties of each concept. Interviewing was stopped when data saturation happened; data saturation occurred when no new codes were identified and when the emerged categories were “coherent.”

###  Trustworthiness

 In the current study, credibility was established by prolonged engagement with participants and member checking. Prolonged engagement with participants in the research area provided the opportunity for deep communication and a better understanding of both participants’ points of view and their work tasks related to the NPPCD. Member checking technique was used to establish the tenet of credibility and to show that the findings are accurate and honest. For member checking, a coded transcript was given to some participants for data revision and to check whether the document matched their perspectives. Besides, selecting participants with diverse experiences increases the possibility of shedding light on the research question from different aspects.^20^ In our study, differences in job tasks, positions, and responsibilities of the participants contributed to a richer variation of the phenomena under study. Dependability and conformability were achieved through an auditing process. Two auditors examined the analytical process and the records of meetings for accuracy; then assessed whether all analytical techniques of the grounded theory had been used. The auditors reviewed the analysis, the descriptive, axial, and selective codes to ensure whether they followed the study data. These auditors were not part of the research team but experts in qualitative research methods. The research team documented all study data and provided a description of the participants and the research process to help the assessment of the present findings’ transferability.

## Results

 After data analysis, two main themes emerged, including (1) implementation barriers and** (**2) inefficient policy-making/planning. According to participants’ statements, the explored barriers at the level of implementation were rooted in the policy-making/planning defects (Figure).

**Figure:**
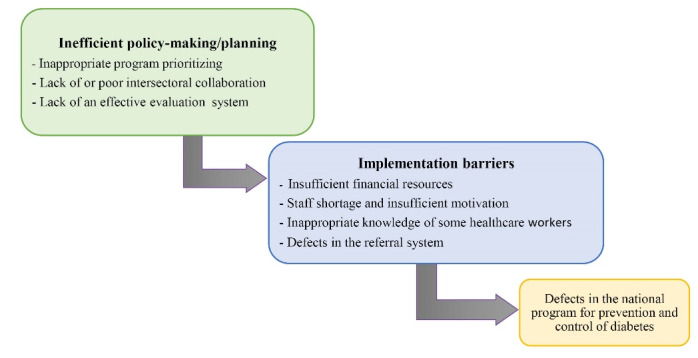
A Conceptual Framework of Barriers to the National Program for Prevention and Control of Diabetes.

 Implementation barriers encompassed four sub-themes: Insufficient financial resources, staff shortage and insufficient motivation, inadequate knowledge of some healthcare workers, and defects in the referral system. Inefficient policy-making/planning included three sub-themes: inappropriate program prioritizing, the lack of or poor intersectoral collaboration, and the lack of an effective evaluation system.

###  Theme 1: Implementation Barriers

 One of the main themes obtained through the data analysis from the participants’ perspectives is the implementation barriers, which interfere with the executive process of the NPPCD. According to the participants’ statements, inefficient policy-making/planning is the leading cause of these obstacles.

####  Insufficient Financial Resources

 A significant number of participants believed that funding shortage for preparing equipment related to screening and monitoring is one of the implementation barriers to the NPPCD. *“Although the devices were purchased and delivered to some healthcare centers, we do not have enough funds to buy kits” *(Healthcare worker).Even though some monitoring equipment is available in healthcare centers, in some cases, the lack of financial resources prevents the provision of all the necessary tools. As a result, sometimes adequate healthcare cannot be provided to manage diabetes. Another participant asserted that*: “Although we have a glucometer in the healthcare centers, sometimes we do not have enough money to buy a strip; therefore, we cannot check patients’ blood glucose” *(Healthcare worker). According to many managers, patients referred to public healthcare centers expect to obtain their medications and access screening or periodic tests at a more reasonable cost. Due to financial problems, some patients refused to have routine check-up tests. *“There is no pharmacy or laboratory in most healthcare centers. Due to the high-cost tests and medications, people no longer come to centers for routine follow-ups. Because they say that if have to spend so much money they prefer to go to a private sector” *(Policy-maker/planner). Accordingly, another participant said the insurance system’s lack of proper coverage of services is another reason for financial problems: *“Patients in big cities and villages near big cities prefer to go to the private sector rather than public healthcare centers. One of the most important reasons is the insurance system’s lack of proper coverage of services” *(Policy-maker/planner).

####  Staff Shortage and Insufficient Motivation

 Many participants in this study noted staff shortage as another obstacle to the optimal implementation of the NPPCD, leading to decreased quality of services or failure to provide necessary services. *“The number of staff is not enough in the healthcare centers” *(Healthcare worker). Some participants believed that the number of physicians, nutritionists, and psychologists working in healthcare centers was insufficient. *“Not all centers have a nutritionist, while one of the principles of diabetes care is to follow a proper diet” *(Healthcare worker). Moreover, healthcare providers have many tasks to do; therefore, they cannot proceed properly with the diabetes care program. Sometimes the busy schedule of healthcare workers causes failure to partake in the diabetes care program. “*We are expected to take care of everything. From vaccination to be a family doctor and family health provider. Then we are also expected to provide diabetes care” *(Healthcare worker). In addition, another point made by the participants was the payment system, which plays a vital role in motivating staff. After implementing the electronic recording system (for recording patients’ information), all payments have been based on the quantity and not the quality of healthcare services. *“The payment system is based on the number of registered services. No one pays attention to the quality of the services” *(Policy-maker/planner).Another participant asserted that:“*One of the problems is that our payment depends on the registered services in the electronic recording system” *(Healthcare worker).

####  Inadequate Knowledge of Some Healthcare Workers

 Another implementation barrier mentioned by the participants was the lack of appropriate knowledge among a group of healthcare workers. Many participants believed that physicians and healthcare providers who started working in public healthcare centers had not received the necessary training and were not sufficiently familiar with health programs and national guidelines. *“Before hiring a doctor at the center, they should be retrained and familiarized with the plans and instructions”* (Policy-maker/planner).Accordingly, another participant described,* “Some healthcare providers are not well-trained. Since diabetes care is challenging, they prefer to do easier tasks” *(Healthcare worker).Additionally, it seems that inadequate knowledge of a group of healthcare workers was not related to the lack of guidelines or protocols. Despite the availability of instructions, some staff refused to follow them. Another participant asserted that:* “Although guidelines and protocols are available, some physicians do not implement them, and it seems Behvarz workers are the only ones who correctly implement programs’ protocols” *(Policy-maker/planner).

####  Defects in the Referral System

 According to the participants, providing diabetes health services at the primary, secondary, and tertiary levels has been one of the goals of the NPPCD. These three levels have been designed for different purposes, including screening, monitoring, treating, and controlling diabetes. Achieving this goal requires a functional referral system. However, due to the malfunction of the referral system, this goal has not been achieved; as a result, people are confused and have unrealistic expectations from the health houses. They assume health houses should provide them with treatment and medical services rather than prevention, screening, and follow-up services.* “People still do not realize that our job is more screening, prevention and follow-up their conditions than treatment” *(Healthcare worker).Several participants stated that another shortcoming of the referral system is that patients receive services from sub-specialized healthcare centers and specialized hospitals through a self-referral process without being referred by the secondary or primary care levels. *“When patients find out they can go to specialized hospitals without being referred by a health house and receive medical services; they are unwilling to come to the health house for the regular follow-up” *(Policy-maker/planner).One of the reasons for providing medical services at three levels is to regulate and facilitate the process of medical services. However, it seems that when a healthcare center refers patients, they encounter malfunctions at the upper levels. *“Sometimes when we refer patients to the hospitals,’ no one guides them in the hospital to do their blood tests or receive other healthcare services” *(Policy-maker/planner).

###  Theme 2: Inefficient Policy-Making/Planning

 Inefficient policy-making/planning is another main theme obtained from reviewing and analyzing the current data. It contains three sub-themes: Inappropriate program prioritizing, the lack of or poor intersectional collaboration, and the lack of an effective evaluation system.

####  Inappropriate Program Prioritizing

 Based on the participants’ statements, the NPPCD has received low priority among the country’s health plans. Accordingly, after implementing new health programs such as the Health Transformation Plan, the NPPCD has been neglected* “Whenever cross-cutting policies, such as policies to prevent and manage diabetes, coincide with national macro-programs, problems happen in the implementation process. Such programs must be integrated and implemented in the same direction. Due to implementing the Health Transformation Plan in healthcare centers, little attention has been paid to the NPPCD” *(Policy-maker/planner). It seems that diverse management models to operate health system programs have disrupted the effective implementation of the NPPCD. Accordingly, another participant said: *“Although the Health Transformation Plan seems to align with the NPPCD, diverse management models prevent these programs from being implemented completely” *(Policy-maker/planner).Financial resource limitations were mentioned as one of the negative consequences of the NPPCD’s low priority in the country’s health system. Inefficient planning in allocating financial resources has caused several problems in the NPPCD’s execution. Another participant revealed: *“One example of inconsistencies in programs prioritizing is funding reduction, especially in the NPPCD, which needs to revitalize funding”* (Policy-maker/planner). Furthermore, human resource shortage could be another disadvantage of the NPPCD’s low priority. A group of managers and experts participating in this study asserted that although there were guidelines for providing adequate staffing, this plan has not been fully implemented. There are not enough healthcare workers in the healthcare centers to prevent and manage diabetes.* “In the initial plan, the number of healthcare workers was appropriately predicted, while now it seems the staff is less than the initial forecast, and the workload is higher than the prediction” *(Healthcare worker).

####  Lack of or Poor Intersectoral Collaboration

 According to the participants, one of the critical problems challenging the NPPCD has been the lack of separation of duties and services at the MoHME and medical universities leading to poor intersectoral collaboration. The lack of efficient intersectoral collaboration wastes financial resources and leads to the implementation of similar projects in the country’s health system. *“The tasks are sometimes overlapped in the medical universities and the ministry of health, and the budget is allocated to several units instead of concentrating on one unit. Moreover, even parallel programs are done in different units due to the lack of intersectoral collaboration” *(Policy-maker/planner).Our participants stated that although there was appropriate communication between healthcare sectors in the early years of implementing the NPPCD, this coordination was discontinued. *“The treatment and healthcare units used to coordinate with each other, but this coordination was lost after a while” *(Policy-maker/planner).

####  Lack of an Effective Evaluation System

 The lack of a constant evaluation system was another critical obstacle to the NPPCD. A group of participants expressed that the necessary evaluations to find the program’s gaps and shortcomings have not been constantly done. According to participants’ statements, an effective evaluation system could improve the program and increase the quality of diabetes healthcare services. *“The diabetes national program was well designed and could have been used as the base of some other health programs, but it was not evaluated after a while” *(Policy-maker/planner).Some participants argued that staff performance should also be evaluated sufficiently in addition to the importance of continuous program evaluation. The evaluation criteria of healthcare center employees should be based on the quality of services, not just being based on the number of services registered in the electronic recording system. An inefficient performance assessment system seems to have led to poor motivation to provide adequate healthcare services. “*Unfortunately, the staff is evaluated based on the number of services registered in the electronic recording system. A health worker may have many service registrations, but in reality, has not served as much, and vice versa” *(Healthcare worker).

## Discussion

 The diabetes prevention and control program in Iran includes 25 years of background. Through these years, along with all changes in the country’s health system, the NPPCD also has faced ups and downs. This study aimed to explore the main barriers to NPPCD from the perspective of policy-makers, planners, and healthcare workers. Based on the participants’ statements, the NPPCD has encountered obstacles in executive and policy-making/planning dimensions. The principal executive barriers were insufficient financial resources, staff shortage and poor motivation, inadequate knowledge of some healthcare workers, and defects in the referral system. These barriers were mainly caused by inappropriate program prioritizing, the lack of or poor intersectoral collaboration, and the lack of an effective evaluation system at the policy-making and planning level.

 The current findings regarding the barriers to the NPPCD align with previous studies from Iran. These studies highlighted that the NPPCD was affected by problems in different domains, including referral system, intersectoral collaboration, financial,^14^ and human resources.^18^ Our findings revealed that high equipment costs, medication, and lack of proper insurance coverage reduced the number of patients referred to public healthcare centers. Similarly, other studies also found that financial problem was a reason for not seeking diabetes care.^21,22^ Zgibor et al^23^ reported that in more than 43% of cases, individuals with diabetes refused diabetes care because of the high cost. Furthermore, a lack of health insurance coverage, especially for diagnostic and advanced services related to the diabetes management program, has been reported in Iran.^14^

 Sufficient and well-trained healthcare workers are critical for a health program’s success.^24^ In the current study, the limited number of healthcare workers, over workload, poor motivation to follow care tasks, and inadequate knowledge of some healthcare workers were some of the implementation barriers. The human-resource-related problems encompassing a shortage of healthcare workers have been identified as one of Iran’s national diabetes program challenges. Due to the shortage of personnel and their busy schedule, patients do not receive necessary medical care in some cases.^18^ Furthermore, despite the importance of adequate health workers’ abundance, the quality of their performance is also critical for presenting proper healthcare.^25^ In this regard, staff motivation could also impact the quality of healthcare services. Since the new job evaluation and payment system is only based on the number of registered services, some healthcare workers do not have enough motivators to deliver quality care, leading to defects in diabetes management. Moreover, efficient healthcare cannot be provided unless health workers are well-trained.^26^ Consequently, insufficient knowledge of some healthcare workers is another hindrance. The lack of appropriate education on diabetes and its national guidelines has created complexities in the healthcare centers. Similarly, in a previous finding, most physicians noticed they need more training to manage diverse aspects of diabetes.^27^

 In agreement with a review from Iran that analyzed the NPPCD’s documents,^14^ our findings showed that the referral system’s defects were a barrier to implementing an effective healthcare program. Leveling the healthcare services to primary, secondary, and tertiary care was a purpose for the NPPCD^12^ that was never met due to the malfunction of the referral system. Since patients could receive tertiary care level services at specialized hospitals and sub-specialized centers without being referred by a primary care level, they have no motivation to attend the health houses for their follow-up care, which leads to complications toward regular monitoring conditions. Consistent with our findings, van Uden et al^28^ found a malfunction in the referral system of the Dutch health system. They reported that patients skip primary care settings and easily access secondary care settings, which causes overcrowding in emergency departments and hospitals.

 Based on the present findings, defects in policy-making and planning encompass inappropriate program prioritizing, the lack of or poor intersectional collaboration, and the lack of an effective evaluation system have been identified as the main reasons for the implementation level’s obstacles. Based on previous studies, factors such as support and prioritizing, intersectoral collaboration, and empowering an evaluation system influence the execution of healthcare programs.^29-31^ According to our results, the healthcare system’s programs need integrated policy-making/planning to achieve public healthcare goals. While the Health Transformation Plan, a national macro program, in some aspects was not along with the NPPCD, leading to neglect in implementing the NPPCD. Accordingly, poor decision-making for program prioritizing caused maladaptive planning and led to lower financial support^32^; since the NPPCD absorbed low priority among the healthcare programs has not received sufficient financial support.

 Intersectoral collaboration among MoHME and different healthcare sectors could affect the healthcare system’s efficacy. As a result, lack of or poor programming for intersectoral collaboration has caused parallel activities and confusion in responsibilities and resource allocation. Based on our participants’ statements, the lack of effective intersectoral collaboration has wasted financial resources and led to several problems at the implementation level. A review from Europe reported that intersectoral collaboration within and outside healthcare systems could improve health promotion activities.^33^ In accord with our findings, a Turkish study emphasized the critical role of policy-making regarding intersectoral cooperation. It acknowledged that achieving this goal needs specific legislation to illustrate tasks and responsibilities.^34^

 Constant program evaluation helps policy-makers and planners to find the existing problems and verify whether new planning or policies are needed.^35^ In our findings, some participants pointed out that the lack of a constant program evaluation caused neglect to monitor the NPPCD. It seems that due to the lack of a constant evaluation system, the policy-makers and planners failed to find the NPPCD’s implementation defects. Besides, effective evaluation protocols for staff performance assessment seem critical, which healthcare workers also mentioned from another perspective. Employees’ performance has been assessed based on only a single method strategy in the current program. While using a multi-method strategy that considers different factors such as knowledge, skills, and attitudes of staff could be more beneficial for evaluating their performance.^36^

 The notable strength of the current study is that for the first time in Iran, we explored the experiences and perceptions of policy-makers and planners in charge of the NPPCD, which could complete previous findings from the perspective of individuals with diabetes and healthcare workers that have already been investigated.^18^ Our results could make an opportunity to compare these results and provide a comprehensive understanding of the NPPCD barriers. Moreover, the number of our participants and their key role in the NPPCD could cause diverse perspectives and practical opinions to solve the program’s problems in the future. To increase the consistency of the data, the principal researcher conducted all interviews. For conformability, coded transcripts were verified by other research team members as well as some of the participants. However, we encountered some limitations; although the perspective of policy-makers and planners was investigated at the national level, the healthcare workers’ perceptions and experiences were explored at the level of rural and urban areas of the Tehran province. Furthermore, another limitation of the present study is not interviewing physicians and other healthcare workers at the tertiary care levels. In this consideration, further research is needed to complete the current findings.

## Conclusion

 Our findings highlighted the main barriers to NPPCD in Iran from the perspective of policy-makers, planners, and healthcare workers. Current results highlighted that inefficient policy-making and planning have led to several implementation problems, including financial limitations, human resource shortage, poor motivation of staff to provide adequate diabetes care, inadequate knowledge of some healthcare workers, and referral system defects. Therefore, effective policy-making and planning focusing on prioritizing the NPPCD, improving intersectoral collaboration, and utilizing a more efficient evaluation system to assess the program and staff performance could be the key strategies to promote this program and provide better diabetes healthcare services.

## Acknowledgements

 The authors would like to express their appreciation to all participants who made this study possible.

## Ethical issues

 The ethics committee of Shahid Beheshti University of Medical Sciences approved this study. Participants were given informed written consent before the interview began.

## Competing interests

 Authors declare that they have no competing interests.

## Authors’ contributions

 SS, MS, FA, PA, and SA conceptualized and designed the study. SS, FM, and PA drafted the manuscript. SS and DK acquired the data. SS, FM, and PA performed the analysis. All authors contributed to the interpretation of the data, the edit and critical revision of the manuscript.
